# Anti-Cholinergic Effects of the Phenolic Extract from the *Astragalus crenatus* Plant: A Computational and Network Pharmacology Study

**DOI:** 10.3390/ph17030348

**Published:** 2024-03-07

**Authors:** Sabrina Lekmine, Ouided Benslama, Hichem Tahraoui, Mohammad Shamsul Ola, Aicha Laouani, Kenza Kadi, Antonio Ignacio Martín-García, Ahmad Ali

**Affiliations:** 1 Biotechnology, Water, Environment and Health Laboratory, Abbes Laghrour University, Khenchela 40004, Algeria; 2 Laboratory of Natural Substances, Biomolecules, and Biotechnological Applications, Department of Natural and Life Sciences, Larbi Ben M’Hidi University, Oum El Bouaghi 04000, Algeria; 3 Laboratory of Biomaterials and Transport Phenomena, University of Medea, Medea 26000, Algeria; 4 Laboratoire de Génie des Procédés Chimiques, Department of Process Engineering, University of Ferhat Abbas, Setif 19000, Algeria; 5 Department of Biochemistry, College of Science, King Saud University, Riyadh 11451, Saudi Arabia; 6 Laboratory of Metabolic Biophysics and Applied Pharmacology, Faculty of Medicine, University of Sousse, Sousse 4002, Tunisia; 7 USCR Analytical Platform UHPLC-MS & Research in Medicine and Biology, Faculty of Medicine, University of Sousse, Sousse 4023, Tunisia; 8 Estación Experimental del Zaidín (CSIC), Profesor Albareda 1, 18008 Granada, Spain; 9 Department of Life Sciences, University of Mumbai, Vidyanagari, Mumbai 400098, India

**Keywords:** *Astraglus crenatus* Schult., Alzheimer’s disease, acetylcholinesterase, butyrylcholinesterase, molecular docking, network pharmacology

## Abstract

Investigations into cholinesterase inhibition have received attention from researchers in recent years for the treatment of Alzheimer’s disease. Cholinesterase enzymes, namely, acetylcholinesterase (AChE) and butyrylcholinesterase (BChE), hold pivotal significance in Alzheimer’s disease (AD) treatment. In this study, we utilized the ethanolic extract of *Astragalus crenatus* followed by liquid chromatography–electrospray ionization tandem mass spectrometry (LC-ESI-MS/MS) to separate and identify at least 21 compounds in the extract. Rosmarinic acid exhibited the highest concentration (96.675 ± 1.3 mg/g extract), succeeded by hesperidin (79.613 ± 1.2 mg/g extract), hesperetin (75.102 ± 1.4 mg/g extract), rutin (68.156 ± 1.6 mg/g extract), chlorogenic acid (67.645 ± 1.5 mg/g extract), fisetin (66.647 ± 2.3 mg/g extract), and hyperoside (63.173 ± 1.5 mg/g extract). *A. crenatus* extract efficiently inhibited both AChE and BChE activities in a dosage-dependent manner. Molecular docking was employed to scrutinize the anticholinesterase mechanisms of the identified phytocompounds. Notably, a network pharmacology analysis was executed for the most efficacious compound. Based on binding energies, hesperidin emerged as the most potent inhibitor against both AChE and BChE, exhibiting scores of −10.5 Kcal/mol and −9.8 Kcal/mol, respectively. Due to its dual inhibition of AChE and BChE activities, hesperidin from *Astragalus crenatus* holds promise for the development of novel therapeutics aimed at neurological disorders, particularly AD.

## 1. Introduction

Alzheimer’s disease (AD) is a chronic and irreversible dementia marked by neurofibrillary tangles and widespread neuronal death [1]. This neurodegenerative disease often begins with minor memory loss and may progress to cognitive deficits, serious behavior issues, or mortality [1]. The development and precise origins of Alzheimer’s disease are still largely unclear; however, the most often advanced arguments include amyloid, metal, and cholinergic activity [2]. The current medication for AD focuses on increasing acetylcholine (ACh) levels by inhibiting cholinesterase enzyme (ChE) activities [2]. Indeed, the current primary treatment strategies for AD are based on targeting the acetylcholine (ACh) level, a critical neurotransmitter for signal transduction.

The neurotransmitter ACh may be hydrolyzed by the enzymes acetylcholinesterase (AChE) and butyrylcholinesterase (BuChE), both of which are found in the neuronal synapses in the central nervous system [3]. Therefore, the use of the bioactive inhibitors acetylcholinesterase (AChE) and butyrylcholinesterase (BChE) is considered to be one of the most effective therapies for the management of neurodegenerative disorders. Reduced symptoms of AD may be achieved by blocking these two enzymes, which improves coordination between the cholinergic system and the nerve terminals [4]. A variety of synthetic drugs, including galanthamine, donepezil, tacrine, memantine, caproctamine, and rivastigmine, have been applied to manage moderate to severe AD [5]. Nevertheless, longer-term trials have demonstrated a deterioration in clinical effectiveness, either due to a loss of pharmacological effectiveness or the inexorable advancement of the disease [4]. In addition, these medications exhibited many adverse effects, including gastrointestinal distress, nausea, vertigo, hepatotoxicity, diarrhea, and vomiting. Given that the currently available AChE inhibitors are not without their flaws, there is a continuing interest in the search for new AChE inhibitors derived from natural sources that have little or no negative effects.

Changes in lifestyle, especially in dietary habits, have arisen as an approach to reducing the incidence of aging and age-associated diseases. There are several plant-derived phenolic compounds with wide-ranging health advantages, and they seem to have neuroprotective properties. Phenolic compounds have gained great attention over the last two decades due to their widespread distribution in many diets and their potent antioxidant activity. Diets high in phenols are regarded as neuroprotective owing to their ability to modulate many cellular pathways related to the etiology of Alzheimer’s disease. In this regard, it has been suggested that intake of plant-based phenolic compounds may prevent cognitive impairment such as AD.

*Astraglus crenatus* Schult. is a member of the family *Fabaceae* (or *Leguminosae*), which is divided into 750 genera and 18,000 species [6]. Plants of the *Astragalus* genus growing in North Africa are native to the Mediterranean and the Arabian Sahara [7,8,9]. They are represented by more than 50 species divided into different divisions, 15 of which can be found in Algeria’s Sahara. In the flora of North Africa, 10 *Astragalus* species are endemic to Algeria, Morocco, and Tunisia. Locally, “Bou akifa” is the name given to the Algerian *A. crenatus*. *Astragalus* species are prized as therapeutic herbs for health problems like stomach ulcers, chronic bronchitis, hypertension, gynecological illnesses, diabetes, and scorpion venomous attacks around the world. Traditional North African medicine has long recognized *A. crenatus* as a particularly toxic species. It makes livestock sick with a condition known as “Asaydal” [10]. It is generally recognized that the *Astragalus* genus has a wealth of biologically active compounds. Prior studies on the phytochemistry of several *Astragalus* plants led to the isolation and characterization of saponins and flavonoids. However, phenolic compounds of *A. crenatus* have never been reported, especially in terms of pharmacological research using in silico approaches. Therefore, in this study, we investigated the potential of this plant and its constituents as a possible treatment for AD. The plant extract was subjected to LC–MS/MS analysis to identify its phenolic compounds [11,12,13]. Additionally, the in vitro anticholinesterase activity of the extract was evaluated against the acetyl and butyryl enzymes. A molecular docking study was carried out in an attempt to better understand the chemical interaction between phenolic inhibitors and the two AChEs and to advocate the consumption of *A. crenatus* or its active phenols as dietary supplements to prevent cognitive deterioration. A network pharmacology study was also performed to determine the probably targeted genes of the bioactive compound, as well as the pathways and biological activities influenced by these genes.

## 2. Results

### 2.1. Identification and Quantification of Phenolic Compounds of A. crenatus

**Figure 1:**
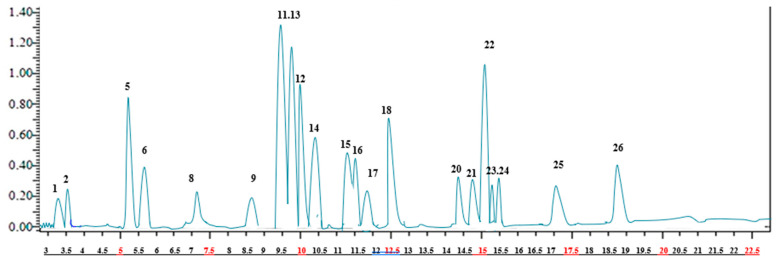
LC-MS/MS chromatogram of the *A. crenatus* ethanolic extract.

The results of the LC-ESI-MS/MS analysis of the *A. crenatus* extract are presented in Figure 1 and Table 1.The analysis revealed the presence of 21 phenolic compounds, with the highest concentration registered for rosmarinic acid (96.675 ± 1.3 mg/g extract), followed by hesperidin (79.613 ±1.2 mg/g extract), hesperetin (75.102 ± 1.4 mg/g extract), rutin (68.156 ± 1.6 mg/g extract), chlorogenic acid (67.645 ± 1.5 mg/g extract), fisetin (66.647 ± 2.3 mg/g extract). hyperoside (63.173 ± 1.5 mg/g extract), 4-hydroxybenzoic benzoic acid (58.184 ± 1.3 mg/g extract), and salicylic acid (55.637 ± 1.3 mg/g extract).

### 2.2. Anticholinesterase Activity

The results mentioned in Table 2 indicate that the *A. crenatus* extract had a remarkable potential to inhibit AChE and BChE in a dose-dependent manner. For the AChE assay, the *A. crenatus* extract showed an IC_50_ value of 7.48 ± 0.23 µg/mL, which was significantly (*p* < 0.05) lower than the positive control galantamine (12.37 ± 1.37 µg/mL), indicating that the ethanol extract was a potent inhibitor of AChE. Furthermore, the extract effectively inhibited BChE with an IC_50_ value of 37.14 ± 0.26 µg/mL, in comparison with the inhibitory effect of galantamine on BChE (IC_50_: 32.16 ± 0.74 µg/mL) (Table 2).

### 2.3. Binding Mode Analysis by the Molecular Docking Approach

**Figure 2:**
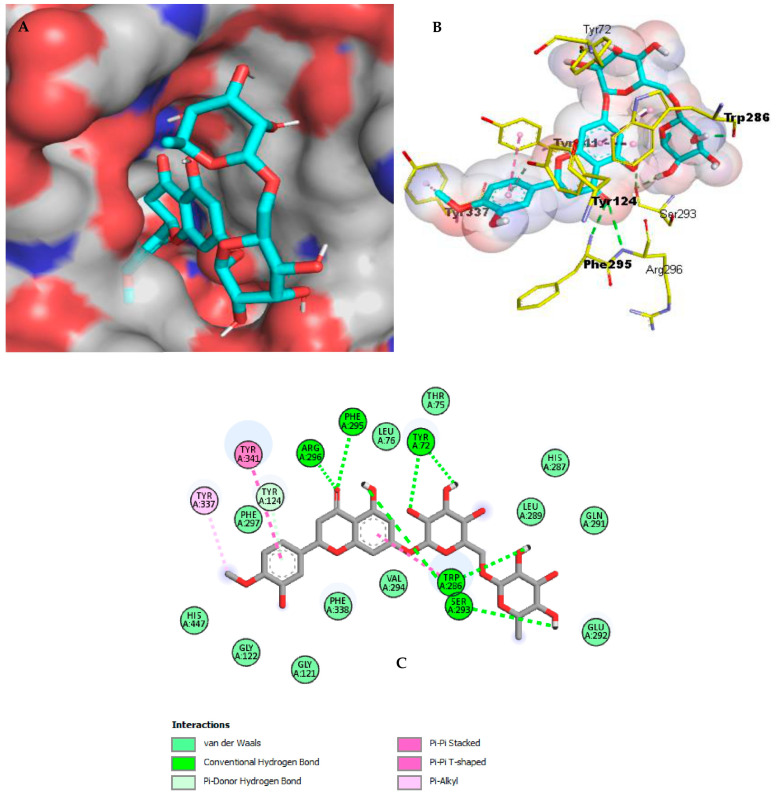
Hesperidin binding mechanism with AChE (**A**) A depiction of hesperidin as cyan sticks attached to the surface binding site of AchE (4EY6). (**B**) Three-dimensional profile of hesperidin as cyan sticks bound in the AchE active site. Key residues are depicted as yellow sticks. (**C**) Two-dimensional illustration of hesperidin interactions with AchE.

**Figure 3:**
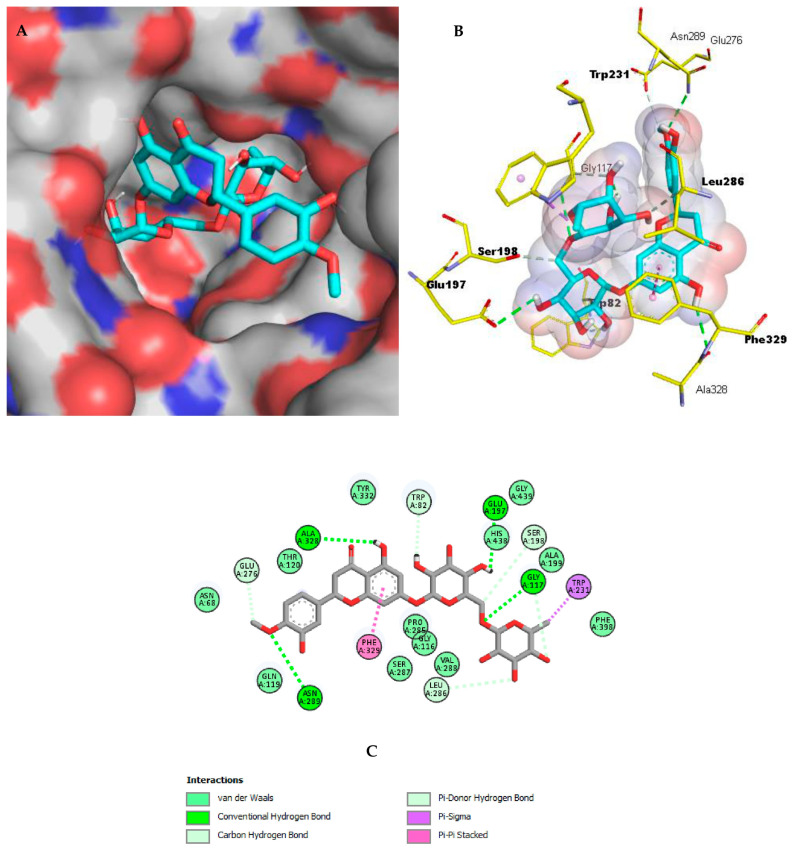
Hesperidin binding mechanism with BchE (**A**) A depiction of hesperidin as cyan sticks attached to the surface binding site of BchE (6EQP). (**B**) Three-dimensional profile of hesperidin as cyan sticks bound in the BchE active site. Key residues are depicted as yellow sticks. (**C**) Two-dimensional illustration of hesperidin interactions with BchE.

The docking of the co-crystallized inhibitors was previously established in order to validate the docking process within the targeted enzymes AChE and BChE. Their inhibitors galantamine and ethopropazine, respectively, exhibited geometric orientations very close to their respective co-crystallized inhibitors with RMSD values of 1.123 and 1.362, respectively, thus confirming the accuracy of the docking configuration. These inhibitors were selected as reference molecules for the in silico study. Only compounds with binding energies equal to or lower than those of the reference inhibitor galantamine were considered as possibly bioactive against the two enzymes. The molecules chosen according to this last criterion are listed in Table 3 and Table 4 and shown in Figure 2 and Figure 3.

### 2.4. Network Pharmacology of the Top Ligand

**Figure 4:**
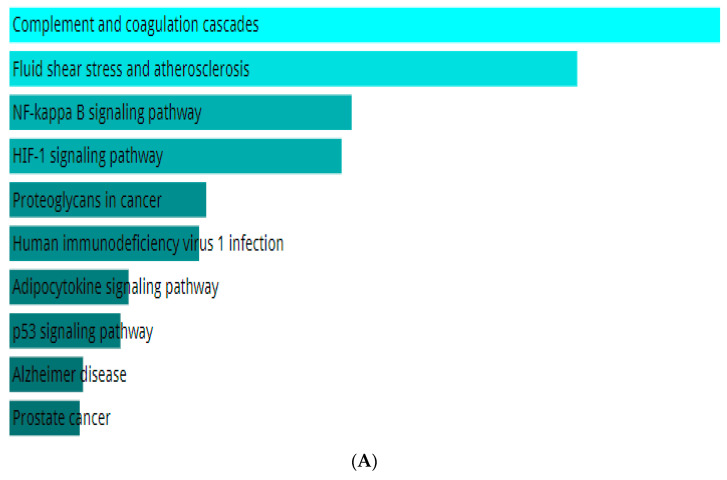

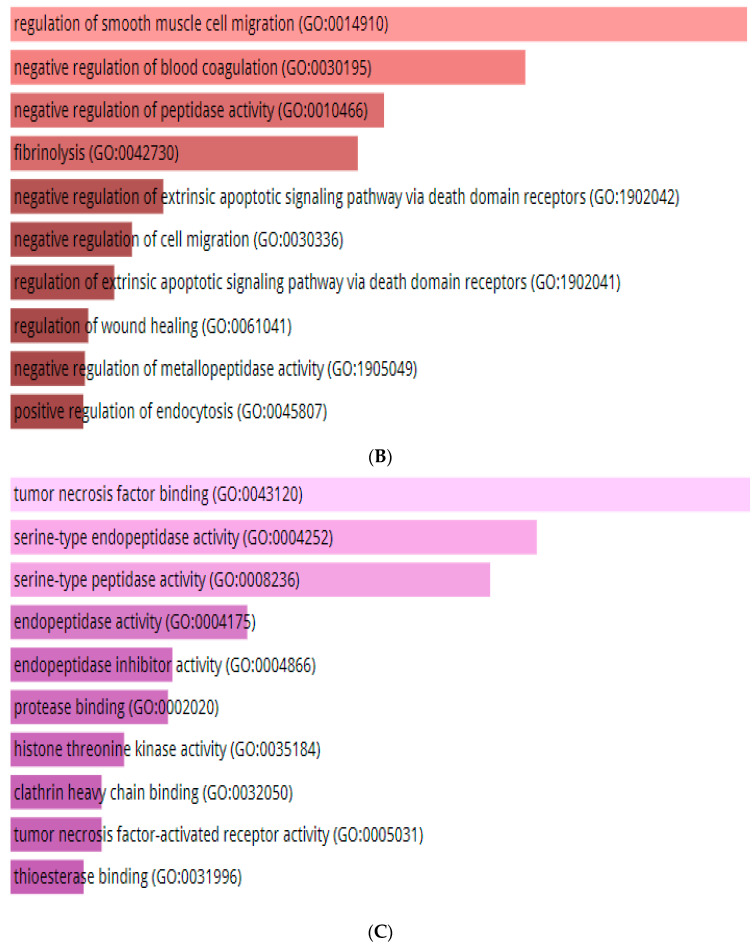

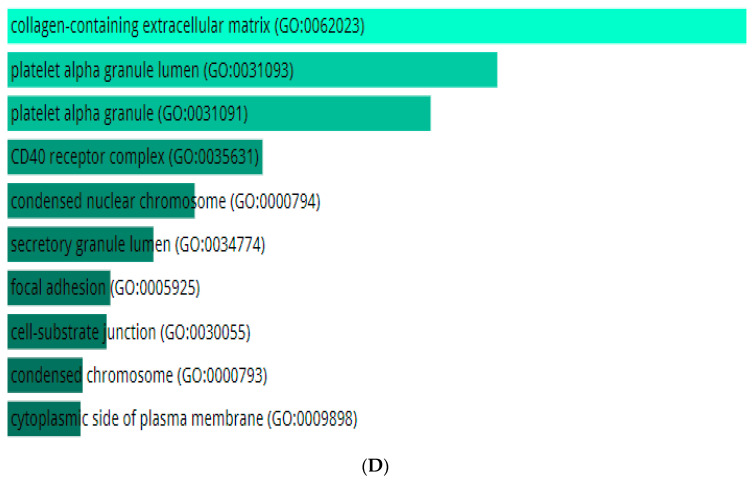
(**A**) KEGG 2021 human genes, (**B**) GO biological process 2021, (**C**) GO molecular functions 2021, and (**D**) GO cellular components 2021.

Enrichment analysis helps us learn about the pathways and biological functions affected by a specific gene set. In this process, the top hesperidin, giving the best results of the in silico inhibition activity toward the targeted enzymes, was tested for enrichment analysis by the Enricher database. The enrichment results listed in Figure 4A indicate that the genes of the KEGG pathway targeted by the top ligand hesperidin are notably associated with complement and coagulation cascades, fluid shear stress and atherosclerosis, the NF-kappa B signaling pathway, the HIF-1 signaling pathway, the adipocytokine signaling pathway, the p53 signaling pathway, prostate cancer, proteoglycans in cancer, the AGE-RAGE signaling pathway in diabetic complications, and human immunodeficiency virus 1 infection. Figure 4B represents the enrichment results of the top 10 GO biological processes, which are the negative regulation of metallopeptidase activity, the regulation of smooth muscle cell migration, the regulation of metallopeptidase activity, death-inducing signaling complex assembly, fibrinolysis, plasminogen activation, cellular response to UV-A, the negative regulation of hemostasis, and replicative senescence. Figure 4C depicts the top 10 GO molecular functions, including tumor necrosis factor binding, histone threonine kinase activity, clathrin heavy chain binding, tumor necrosis factor-activated receptor activity, thioesterase binding, death receptor activity, tumor necrosis factor receptor binding, metalloendopeptidase inhibitor activity, serine-type endopeptidase activity, and serine-type peptidase activity. The GO cellular components affected by the enrichment process are the CD40 receptor complex, platelet alpha granule lumen, condensed nuclear chromosome, platelet alpha granule, collagen-containing extracellular matrix, condensed chromosome, cytoplasmic side of the plasma membrane, tertiary granule membrane, nuclear chromosome, and specific granule membrane (Figure 4D).

**Figure 5:**
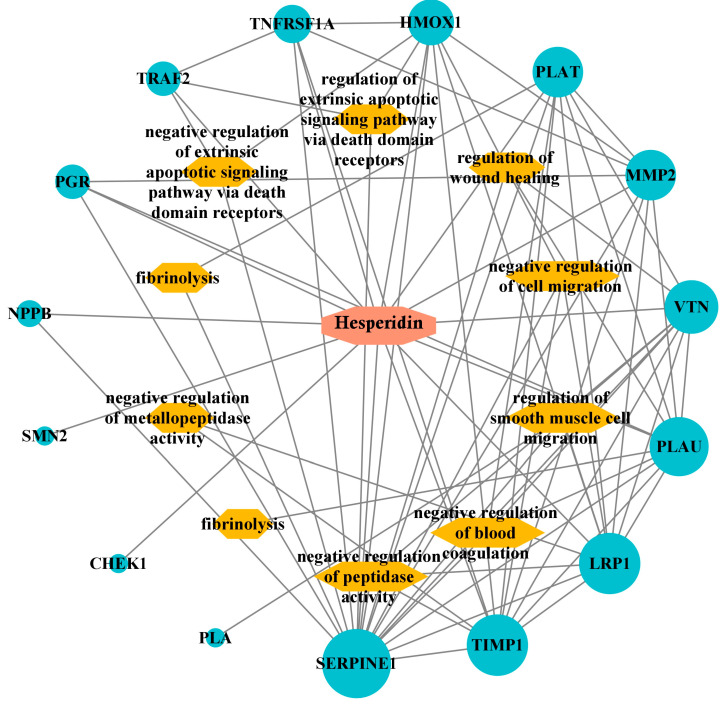
Hesperidin targets pathway network.

To visualize the interactions between the top ligand of this study, hesperidin, its targeted genes, and their biological process, a network of interactions was constructed by importing the drug candidate hesperidin, 15 target genes, and the top 10 pathways into the Cytoscape program, as shown in Figure 5. This network provides insight into the existing genetic relationships of the best active phytocompound and helps to determine the potential functions of these genes. The top ligand hesperidin appears as a red octagon, the blue ellipses are the potential targeted genes, and the orange hexagons are their predicted pathways. A node is depicted by degree value, and the node size is proportional to degree value.

## 3. Discussion

The phenolic substances detected in *A. crenatus* extract can be organized into three distinct groups: (1) phenolic acids, (2) phenolic aldehyde, and (3) flavonoids (flavanol, flavone, flavonol–glycoside, flavones–glycoside, methylated flavonol, flavanone, and flavonone glycoside). In terms of the phenolic acids group, rosmarinic acid, the compound with the highest concentration in this group, is characterized as an ester formed from caffeic acid and 3,4-dihydroxyphenyllactic acid. This compound exhibits various biological activities, such as antioxidant, anti-inflammatory, and neuroprotective effects, as previously documented [14]. Furthermore, chlorogenic acid emerged as the compound with the second-highest concentration in this group. It belongs to the category of cinnamate esters, formed through the formal condensation of the carboxy group of *trans*-caffeic acid with the 3-hydroxy group of quinic acid. Significantly, this acid functions as an intermediate metabolite in lignin biosynthesis and plays essential roles as both a plant metabolite and a component commonly found in food. Furthermore, it acts as a conjugate acid of chlorogenate. Notably, chlorogenic acid has been studied in trials related to the treatment of advanced cancer and impaired glucose tolerance [14].

The third compound with the highest amount was identified as 4-hydroxybenzoic acid, also known as monohydroxy benzoic acid. This compound is a derivative of benzoic acid with a hydroxy substituent located at position C-4 on the benzene ring. Its primary function includes serving as a metabolite in both plants and algae. Furthermore, it acts as the conjugate acid of a 4-hydroxybenzoate compound and finds application as a preservative in cosmetics and certain ophthalmic solutions [14].

Among the isomers of hydroxybenzoic acid, salicylic acid, also known as 2-hydroxybenzoic acid, is present in this extract as well [15]. It ranks as the fourth most abundant compound in the phenolic acid group in terms of concentration. Salicylic acid serves multiple purposes as it is utilized as a food preservative and antiseptic. Its medicinal properties have been recognized for a considerable period, particularly for its ability to combat fever. In addition, salicylic acid is a common component in numerous dermatology products, often combined with other active ingredients. Notably, it is used to treat conditions such as acne, warts, and hyperhidrosis [15]. Protocatechuic acid (PCA) was also identified in the same extract. PCA contains hydroxy groups positioned at positions 3 and 4 on the benzene ring. This compound serves as a human xenobiotic metabolite and a plant metabolite, and it acts as an antineoplastic agent [15]. PCA is a significant metabolite of antioxidant polyphenols commonly found in green tea. Its effects on normal and cancer cells have been investigated through in vitro and in vivo studies, and the results show a mix of outcomes. In addition to the aforementioned compounds, the extract contains three other phenolic acids: malic acid, *trans*-caffeic acid, and quinic acid. These three compounds were detected in the extract at a moderate concentration [16,17,18].

Considering the phenolic aldehyde group, it becomes evident that the extract contains only one substance: vanillin. Vanillin belongs to the class of benzaldehydes, characterized by methoxy and hydroxy substituents at positions 3 and 4, respectively [19]. This compound exhibits versatility as it serves as a plant metabolite and fulfills several roles, functioning as an anti-inflammatory agent, an antioxidant, and an anticonvulsant [19]. Regarding the flavonoid compounds group, the observed substances can be categorized into various subgroups based on the presence of the hydroxy group, methoxy group, hydrogen, and attached glycoside. Based on this deduction, we can establish the first subgroup known as flavones ref comprising two compounds, luteolin and apigenin, both of which were detected in the extract at a significant concentration. Besides this, several flavone molecules substituted by the hydroxy group were detected as flavonols, such as Myricetin (3, 3′, 4′, 5, 5′, 7-hexahydroxyflavone), fisetin (3, 3′, 4′, 7-tetrahydroxyflavone), and kaempferol (3, 5, 7, 4′-tetrahydroxyflavone). In additionally to flavonol compounds, two types of quercetin were detected in this extract: quercetin (3, 3, 4′, 5, 7-pentahydroxyflavone) and rhamnetin or monomethoxyflavone, which is quercetin methylated at position 7 [20].

In terms of the associated glycosides, the subgroup comprises rutin and hyperoside. These compounds serve as both metabolites and antioxidants and were detected in the plant tested at high concentrations [21]. Hesperetin, hesperidin, and naringenin belong to the flavanone subgroup. These compounds are typically glycosylated by a disaccharide, forming flavanone glycosides [21]. Multiple studies have identified phenolic acid and flavonoid constituents in different *Astragalus* species. These compounds play a crucial role in endowing the plant with its antioxidant capacity and various other biological functions [21]. Indeed, our research strongly confirms that within the *Astragalus* genus, flavonoids hold a prominent position as the most abundant category of secondary metabolites. Moreover, the phenolic compounds mentioned earlier in our research were discovered in the methanol extract of *A. schizopterus* [22]. Similarly, the key phenolic compounds, including kaempferol, quercetin, rutin, and rosmarinic acid, were identified in separate *Astragalus* species using two HPLC techniques [22].

The comprehensive phenolic profiles of *Astragalus quisqualis* and *Astragalus kabadianus,* which are extensively documented in the studies conducted by Yasinov [23] and Yasinov [24], respectively, are remarkably similar to those of our species. Comparing our research results with those of the same type of plant is unnecessary, as our study stands as the inaugural investigation of this particular plant species. Numerous variables, such as ecological and climatic conditions, genotypes, geographic locations, and environmental stress on the investigated plant, can account for the variance in chemical composition among members of this genus [25].

Alzheimer’s disease (AD) is a chronic neurodegenerative disorder with different etiological factors [26]. Currently, there are no drugs available to cure AD or any of the other common types of dementia. However, two approaches have been developed to treat AD: preventing the onset of the disease by targeting the primary progenitors and providing symptomatic treatment to protect against cognitive decline [26]. Presently, only three cholinesterase inhibitors (donepezil, galantamine, and rivastigmine) have been approved by the FDA to treat AD. Cholinesterase inhibitors are the only current treatment options available for AD, but they have limited effectiveness and may cause various toxic effects [27]. As the current treatments for Alzheimer’s disease (AD) have limitations, researchers have turned to exploring natural sources like plants to discover new drugs. In this study, we investigated the potential of *A. crenatus* extract to inhibit AChE and BChE activities in a dose-dependent manner, and our results indicate significant inhibitory effects, as demonstrated in Table 2. Various natural compounds belonging to different chemical classes have been reported to exhibit inhibitory effects on acetylcholinesterase and butyrylcholinesterase enzymes. Several studies have demonstrated the potential of polyphenols as potent anti-cholinesterase agents [28]. In a recent study by Liu [20], the authors demonstrated significant anti-Alzheimer’s disease potential by testing a variety of bioactive compounds from *A. membranaceus*, which was also confirmed by molecular docking targeting the AChE enzyme. On the other hand, we successfully highlighted the anti-AChE activity of the *A. armatus* extract by obtaining an IC_50_ of 40.25 ± 1.41 g/mL [6]. Anti-cholinesterase activity was also demonstrated for the methanolic extract of the *A. alopecurus* species with an IC_50_ of 1.99 ± 0.9923 µg/mL compared with the standard tacrine, which showed a value of 0.0246 ± 0.9706 µg/mL [29]. In another study, *A. dumanii* showed interesting anti-cholinesterase potential with IC_50_ values of 1.47 µg/mL for AChE and 0.83 µg/mL for BChE compared with the standard tacrine, which showed IC_50_ values of 19.11 µg/mL and 12.36 µg/mL for the two enzymes, respectively [30].

The computational investigation conducted in this study aimed to explore the anti-cholinergic effects of phenolic extracts from *A. crenatus,* focusing on their potential as inhibitors for acetylcholinesterase (AChE) and butyrylcholinesterase (BChE) in the context of Alzheimer’s disease (AD) treatment. This study integrated molecular docking to elucidate the binding modes, stability, and dynamics of the interactions between the identified compounds and the target enzymes [31].

Based on the docking results, galantamine showed a binding energy of −9.8 kcal/mol when interacting with AChE. Eight compounds depicted scores equal to or lower than this value, the best of which were hesperidin (−10.5 kcal/mol), luteolin (−10.4 kcal/mol), rosmarinic acid (−10.3 kcal/mol), and apigenin (−10.3 kcal/mol). The binding mode analysis for BChE included the evaluation of co-crystallized and reference inhibitors, with galantamine exhibiting a binding energy of −9.3 kcal/mol. Ten molecules were selected based on their binding energies, and the top three ligands, i.e., hesperidin, rhamnetin, and rutin, showed promising results with binding energies ranging from −9.7 to −9.8 kcal/mol.

The key amino acids composing the active sites of the two enzymes AChE and BChE are well defined in crystallographic resolution studies of their 3D structures. These key residues can be clearly identified from the interactions with the co-crystallized inhibitors that are given in Table 3 and Table 4. The conserved amino acids of the active site gorge that have been reported in the literature for AChE/BChE, respectively, include the catalytic and oxyanion hole residues Ser203/Ser198 and His447/His438; choline-binding pocket residues, which are Glu202/Glu197, Tyr337/Ala328, and Trp86/Trp82; and acyl-binding pocket residues, which are Trp236/Trp231, Phe338/Phe329, Phe297/Val288, and Phe295/Leu286 [32]. All the compounds selected as possible inhibitors of the two ChEs form interactions with at least one of their key amino acids (Table 3 and Table 4).

In the case of AChE, the best ligand hesperidin generates eight hydrogen bonds, three of which are formed with the peripheral anionic subsite residues Tyr124 (3.65 Å), Trp286 (3.02 Å), and Trp286 (1.93 Å) and one with the acyl-binding pocket residue Phe295 (2.84 Å). In addition, two Pi-Pi Stacked interactions are established with Tyr341 and Trp286 and the benzene rings of hesperidin, while one Pi-Alkyl interaction is produced with the Tyr337 residue composing the peripheral anionic subsite of AChE. Furthermore, van der Waals attractions are generated with a variety of amino acids, including the acyl-binding pocket residues Phe338 and Phe297 (Figure 3). Thus, with a total of eight hydrogen interactions, hesperidin affords robust binding into the catalytic pocket of AChE.

Similarly, hesperidin was the best ligand in the case of BChE. Hesperidin mediates nine hydrogen bonds with the catalytic site of BChE; among them, one is formed with the oxyanion hole residue Ser198 (3.27 Å), three with choline-binding pocket residues Glu197 (2.77 Å), Trp82 (2.33 Å), and Ala328 (2.76 Å), and one with the acyl-binding pocket residue Leu286 (3.66 Å). This ligand also interacts by forming Pi-Sigma and Pi-Pi Stacked hydrophobic bonds with the acyl-binding pocket residues Phe329 and Trp231, respectively. Several van der Waals interactions are also established, increasing, therefore, the firmness and the stability of the BChE–hesperidin complex (Figure 4).

From the molecular docking results, it is noticed that the tested phenolic compounds docked differently within the two ChEs. Some compounds gave binding energies allowing for their designation as possible inhibitors for one ChE but not for the other. This is the case, for example, of rosmarinic acid, apigenin, and naringenin, which are well-docked for AChE but less efficient for BChE. Similarly, only BChE demonstrated acceptable docking scores for rhamnetin, rutin, hyperoside, fisetin, and kaempferol. The discrepancies in the binding affinity of these ligands within the two enzymes’ active site gorges are most likely related to differences in their two acyl-binding pockets, where the bulky amino acids Phe297 and Phe295 of AChE are substituted by the two small amino acids Val288 and Leu286 in the cavity of BChE, thus offering a more spacious volume in its pocket for a wider possibility of anchoring [33]. Besides this specific selectivity, another category of compounds docked quite well for the two ChEs; this was the case, for example, of hesperidin, hesperetin, myricetin, quercetin, and luteolin. These ligands are, therefore, considered nonselective or dual AChE and BChE inhibitors.

## 4. Materials and Methods

### 4.1. Plant Material

*A. crenatus* seeds were gathered in Biskra. The GPS coordinates of Biskra, Algeria, are approximately 34.8483° N latitude and 5.7269° E longitude. The altitude of Biskra is approximately 89 m above sea level. The seeds were grown in 3 kg pots. After 4 weeks of germination, the seedlings were transplanted into fresh pots (one seedling at a time) in the controlled environment of the greenhouse, utilizing irrigation at half-field capacity. The seedlings were pruned in June after germination and allowed to dry naturally. Using a micro-fine crusher, the plants were crushed into a powder and kept until needed.

### 4.2. Preparation of Plant Extract

From 10 g of plant powder, the hydro-alcoholic extract (ethanol 70/30) was obtained, and the mixture was macerated for 24 h at room temperature. The filtrate was then concentrated using rotavapor technology (HAHNVAPOR) at 40 °C under a vacuum of 40 millibars.

### 4.3. Equipment and Chromatographic Parameters

A tandem MS system in conjunction with the UHPLC (Nexera type Shimadzu, Columbia, MD, USA) was used for the LC–MS/MS technique [34]. Furthermore, the LC–MS analysis was also performed using LC-30AD binary pumps, a CTO-10ASvp column oven, a DGU-20A3R degasser, and a SIL-30AC autosampler, and the separation was carried out using a reversed-phase C18 Inertsil ODS-4 analytical column of 150 mm × 4.6 mm, 3 μm at 40 °C. Mobile phase A (H_2_O, ammonium formate (5 mM), and formic acid 0.1%) and mobile phase B (methanol, ammonium formate (5 mM), and formic acid 0.1%) were used in the elution gradient. The solvent flow rate was kept at 0.5 mL/min, and the injection was fixed at 4 μL.

### 4.4. Cholinesterases Inhibition

To evaluate the ability of the *A. crenatus* extract to inhibit the activity of acetylcholinesterase (AChE) and butyrylcholinesterase (BChE), we conducted the Ellman assay [35]. Acetylthiocholine iodide and butyryl thiocholine chloride were used as substrates for AChE and BChE activity, respectively. DTNB (5,5′-Dithiobis[2-nitrobenzoic-acid]) was employed as the cholinesterase activity indicator. To initiate the reaction, 10 μL of 0.5 mM DTNB in sodium phosphate buffer (100 mM, pH 8.0), 40 μL of the *A. crenatus* extract of different concentrations, and 20 μL of the enzyme solution (either AChE or BChE) were mixed and incubated for 15 min. The reaction was started by adding 40 μL of the respective substrates (acetylthiocholine or butyryl thiocholine) for each enzyme. The hydrolysis of the substrates was monitored at 412 nm, and the formation of the yellow 5-thio-2-nitrobenzoate anions indicated the reaction of thiocholine with DTNB. Galantamine was used as standard compound. The IC_50_ values were determined as the concentration of the sample that inhibited half of the hydrolysis of acetyl and butyryl thiocholine. The inhibition percentage was determined using the formula:

$$I\left(\%\right)=\frac{E-S}{E}\;\times 100$$

where *E* represents the enzyme activity in the absence of any test sample, while *S* represents the enzyme activity in the presence of the test sample.

### 4.5. Statistical Analysis

The statistical analysis of the data was performed using GraphPad Prism Data Editor 6.0. One-way ANOVA was used to analyze the data, followed by Dunnet’s test to determine significant differences between the test and control groups. Analysis was performed in triplicate and the results were presented as mean ± standard error of the mean. A *p*-value of less than 0.05 was considered statistically significant.

### 4.6. Molecular Docking Protocol

The 3D structures of AChE and BChE were obtained from the Protein Data Bank (PDB) with the identifiers ID: 4EY6 and 6EQP, respectively. The structures of the 21 phenolic compounds of *A. crenatus* were obtained in SMILES format from the PubChem database and then transferred to 3D structures using the Chimera 1.15 program. Receptors and ligands structures were prepared using Chimera tools by removing co-crystallized heteroatoms, adding hydrogen atoms and a Gasteiger charge, and minimizing their energy. Autodock Vina was used as a molecular docking engine by specifying a grid box around the protein’s active sites where co-crystallized inhibitors are located. For the validation of the docking process, the co-crystallized inhibitors galantamine, and ethopropazine of the respective proteins AChE and BChE were selected as the first ligands to be docked. The visualization of the established interactions within the docked receptor–ligand complexes was achieved using the PyMOL and Discovery Studio packages.

### 4.7. Network Pharmacology of the Top Ligand

The phenolic compound giving the most promising results with the two targeted proteins was the subject of a prediction study of their Network Pharmacology. First of all, the predicted targeted proteins of the top ligand were identified by the freely available web service Way2drug “http://www.way2drug.com/GE/ (accessed on 10 December 2023)” [36] with a probable activity (Pa) of 0.5. Functional enrichment analysis of the predicted target proteins was then performed using the STRING program (V.11.0) “https://string-db.org/ (accessed on 10 December 2023)” [37] to generate a protein–protein interaction network. The proteins of the latter network underwent a second enrichment by submitting them to the Enricher database “https://maayanlab.cloud/Enrichr/ (accessed on 10 December 2023)” to carry out a pathway enrichment analysis for the selected genes using the Kyoto Encyclopedia of Genes and Genomes (KEGG) and to study their Gene Ontology (GO) cellular component, GO molecular function, and GO biological process. Finally, the network between the top ligand and its targets was constructed using Cytoscape (V.3.8.2.) software [38].

## 5. Conclusions

*A. crenatus* extract efficiently inhibited both AChE and BChE activities in a dosage-dependent manner. The in silico screening confirmed the anticholinergic activity of *A. crenatus* phenols by acting as inhibitors of AChE and BChE enzymes. Due to their natural origin, the phenols of *A. crenatus* have a high level of safety and represent a valuable nutritional supplement for preventing Alzheimer’s disease. We proved that the phenolic compounds of *A. crenatus*, in particular, hesperidin with a binding energy of −10.5 kcal/mol and −9.8 kcal/mol with AChE and BChE, respectively, may be proposed as novel food-derived molecules with anti-AD activity. Extensive investigations with in vivo models would be required to develop a better understanding of the pharmacokinetic characters of the promising phenols in a biological system set. The large data set produced from the in silico molecular modeling offers significant training data that help to develop a future food by-product of *A. crenatus* to prevent AD.

## Figures and Tables

**Table 1:** Phenolic compounds detected in the *A. crenatus* extract.

Compound		(*m/z*)	MS2	Quantification (mg Analyte/g Extract)
1	**Quinic acid**	191.0	85 (22), 93 (22)	0.221 ± 2.3 g
2	**Malic acid**	133.1	115 (14), 71 (17)	0.334 ± 2.3 i
3	**tr-Aconitic acid**	172.9	85 (12). 129 (9)	N.D.
4	**Gallic acid**	169.1	125 (14), 79 (25)	N.D.
5	**Chlorogenic acid**	353.0	191 (17)	67.645 ± 1.5 a
6	**Protocatechuic acid**	153.0	109 (16), 108 (26)	39.986 ± 2.2 b
7	**Tannic acid**	183.0	124 (22), 78 (34)	N.D.
8	***trans*-Caffeic acid**	179.0	135 (15), 134 (24), 89 (31)	0.256 ± 2.3 i
9	**Vanillin**	151.1	136 (17), 92 (21)	0.558 ± 1.4 h
10	**p-Coumaric acid**	163.0	119 (15), 93 (31)	N.D.
11	**Rosmarinic acid**	358.9	161 (17), 133 (42)	96.675 ± 1.3 a
12	**Rutin**	609.1	300 (37), 271 (51), 301 (38)	68.156 ± 1.6 g
13	**Hesperidin**	611.1	303, 465	79.613 ± 1.2 c
14	**Hyperoside**	463.1	300, 301	63.173 ± 1.5 c
15	**4-OH Benzoic acid**	137.0	93, 65	58.184 ± 1.3 b
16	**Salicylic acid**	137.0	93, 65, 75	55.637 ± 1.3 h
17	**Myricetin**	317.0	179, 151, 137	4.158 ± 1.5
18	**Fisetin**	285.0	135, 121	66.647 ± 2.3 h
19	**Coumarin**	147.0	103, 91, 77	N.D.
20	**Quercetin**	300.9	179, 151, 121	7.853 ± 1.1 d
21	**Naringenin**	271.0	151, 119, 107	6.253 ± 1.1 de
22	**Hesperetin**	301.0	164, 136, 108	75.102 ± 1.4 h
23	**Luteolin**	285.0	175, 151, 133	4.291 ± 1.3 e
24	**Kaempferol**	285.0	217, 133, 151	5.461 ± 1.4 ef
25	**Apigenin**	269.0	151, 117	25.147 ± 1.6
26	**Rhamnetin**	315.0	165, 121, 300	35.658 ± 2.3 h
27	**Chrysin**	253.0	143, 119, 107	N.D.

Values with different letters in the same columns indicate statistically significant differences (*p* < 0.05).

**Table 2:** IC_50_ values of the *A. crenatus* extract and the standard galantamine in AChE and BChE inhibitory activity assays.

Samples	AChE IC_50_ (μg/mL)	BChE IC_50_ (μg/mL)
Galantamine	12.37 ± 1.37	32.16 ± 0.74
*A. crenatus* extract	7.48 ± 0.23	37.14 ± 0.26

**Table 3:** The best results for the docking of *A. crenatus* phenolic ligands with the AChE target.

Compounds	Binding Energy (kcal/mol)	Hydrogen Interactions (Distance in Å)	Hydrophobic Interactions	van der Waals Interactions
**Galantamine**	−9.8	Ser203 (2.91 Å), Glu202 (2.65 Å), His447 (3.58 Å), Asp74 (3.72 Å), Tyr124 (3.46 Å)	Tyr337, Trp86, Gly121, Phe338, Phe295, Phe297, His447	Gly122, Ser125, Tyr341, Gly120, Tyr133, Gly448
**Hesperidin**	−10.5	Tyr124 (3.65 Å), Arg296 (3.27 Å), Phe295 (2.84 Å), Tyr72 (2.91 Å), Tyr72 (1.98 Å), Trp286 (3.02 Å), Trp286 (1.93 Å), Ser293 (2.08 Å)	Tyr341, Tyr337, Trp286	Glu292, Gln291, Leu289, His287, Thr75, Leu76, Phe297, His447, Gly122, Gly121, Phe338, Val294
**Luteolin**	−10.4	Phe295 (2.84 Å), Arg296 (2.93 Å), Tyr124 (3.43 Å), Tyr124 (4.19 Å), Gly122 (2.88 Å)	Trp286, Tyr341	Tyr337, His447, Ser203, Gly121, Ala204, Phe338, Phe297, Val294, Ser293
**Rosmarinic acid**	−10.3	Ser203 (2.92 Å), Glu202 (2.79 Å), His447 (2.23 Å), Asp74 (2.58 Å), Tyr337 (2.93 Å), Tyr124 (3.04 Å), Tyr341 (2.88 Å), Arg296 (2.40 Å), Ser293 (2.9 Å 3)	Trp86, Tyr341	Trp439, Phe295, Trp286, Phe297, Val294, Gly121, Phe338, Gly122, Ala204, Gly448
**Apigenin**	−10.3	Ser203 (2.96 Å), Tyr124 (3.51 Å)	Tyr341, Trp286	Ala204, Gly121, His447, Ser293, Val294, Phe297, Phe338, Tyr337, Phe295, Arg296
**Hesperetin**	−10.2	Ser203 (3.04 Å), Tyr124 (2.96 Å), Phe295 (2.82 Å), Arg296 (2.16 Å)	Phe338, Tyr124, Trp286	Val294, Tyr341, Tyr337, Phe297, Ala204, Gly121, His447
**Myricetin**	−10.0	Trp86 (2.82 Å), Asn87 (2.67 Å), Tyr133 (3.03 Å), Ser125 (3.68 Å)	Tyr124, Trp86	Tyr72, Tyr341, Tyr337, Gly448, His447, Ile451, Gly120, Ser203, Ser125, Pro88, Gly126, Val73, Gln71
**Quercetin**	−9.8	Tyr72 (3.06 Å), Tyr341 (2.91 Å), Tyr124 (3.53 Å), Val294 (3.22 Å)	Tyr341, Trp286	Ser293, Arg296, Phe297, Phe338, Gly122, Gly121, His447
**Naringenin**	−9.8	Ser203 (2.53 Å), Tyr124 (3.13 Å)	Phe338, Trp286, Tyr341	Tyr337, His447, Gly121, Gly120, Ala204, Phe297, Val294, Ser293, Arg296, Phe295

**Table 4:** The best results for the docking of *A. crenatus* phenolic ligands with the BChE target.

Compounds	Binding Energy (kcal/mol)	Hydrogen Interactions (Distance Å)	Hydrophobic Interactions	van der Waals Interactions	Electrostatic Interactions
**Ethopropazine** **(co-crystallized)**	−8.9	-	Trp82, Tyr332, Gly116, Phe329, Leu286, Trp231	Phe398, Ser198, Trp430, Ser79, Thr120, Gln119, Ser287, Gly117, Pro285, Val288	Asp70
**Galantamine**	−9.3	Trp82 (3.58 Å), Gly116 (3.62 Å), Thr120 (2.16 Å)	Trp82, Leu125	Ser79, Met437, Tyr440, Ala328, Trp430, His438, Gly115, Gly121, Asp70	Trp82
**Hesperidin**	−9.8	Glu276 (3.50 Å), Asn289 (3.08 Å), Leu286 (3.66 Å), Gly117 (3.03 Å), Gly117 (2.99 Å), Ser198 (3.27 Å), Glu197 (2.77 Å), Trp82 (2.33 Å), Ala328 (2.76 Å)	Phe329, Trp231	Phe398, Ala199, Gly439, Tyr332, Thr120, Gln119, Pro285, Gly116, Ser287, Val288	
**Rhamnetin**	−9.7	Ser198 (2.77 Å)	Leu289, Trp231, Phe329, Trp430, Tyr440, Met437, Trp82, Ala328, His438	Ser287, Val288, Phe398, Gly117, Ala199, Gly115, Glu197, Gly439	
**Rutin**	−9.7	Asn289 (3.68 Å), Tyr440 (3.85 Å), Gly78 (4.28 Å), His438 (3.65 Å)	Tyr332, Trp82, Ala328, Phe329	Asp70, Pro285, Phe398, Leu286, Gly117, Ala277, Asn68, Val288, Gln119, Gly439, Met437, Trp430, Ser79	-
**Hyperoside**	−9.5	Ser198 (3.15 Å), Glu197 (2.15 Å), Glu197 (2.55 Å), Tyr128 (2.55 Å), His438 (1.63 Å), Tyr440 (2.94 Å), Gly78 (2.83 Å), Thr120 (3.13 Å), Gly116 (3.22 Å)	Ala328, Trp82	Ala199, Gly115, Ile442, Tyr114, Gly439, Met437, Trp430, Ser79, Tyr332, Phe329, Gly117	Asp70
**Fisetin**	−9.5	Ser198 (1.98 Å), Gly117 (2.87 Å), Gly116 (2.98 Å)	Leu286, Trp231, Phe329, His438, Trp82	Phe398, Ala328, Trp430, Glu197, Gly115, Ala199, Ser287, Pro285, Val288	
**Quercetin**	−9.5	Gly117 (2.89 Å), Ser287 (3.12 Å), Leu286 (3.08 Å)	Trp82, Trp231, Phe329, His438, Leu286	Pro285, Ala199, Ser198, Gly115, Glu197, Gly439, Ala328, Phe398	-
**Hesperetin**	−9.4	His438 (2.96 Å), Glu197 (2.38 Å), Thr120 (3.78 Å)	Trp82	Gly439, Ile442, Tyr128, Gly115, Gly116, Pro84, Ile69, Asn68	Asp70
**Kaempferol**	−9.4	Ser198 (2.83 Å)	Phe329, Leu286, Trp231, Gly116, His438, Trp82	Val288, Phe398, Ala199, Gly115, Glu197, Met437, Ala328	
**Luteolin**	−9.4	His438 (3.10 Å), Tyr128 (2.12) Å, Thr120 (4.85 Å)	Trp82	Ile442, Gly115, Gly116, Pro84, Asn83, Ile69, Asn68, Gly439	Asp70
**Myricetin**	−9.3	His438 (2.66 Å), Tyr128 (3.52 Å)	Trp82	Ser79, Gly439, Ile442, Gly115, Gly116, Gly116, Tyr114, Pro84, Asn83, Ile69, Asn68	Asp70

## Data Availability

Data is contained within the article.
